# High-Sensitivity Seawater Refraction Index Optical Measurement Sensor Based on a Position-Sensitive Detector

**DOI:** 10.3390/s24072050

**Published:** 2024-03-23

**Authors:** Guanlong Zhou, Liyan Li, Yan Zhou, Xinyu Chen

**Affiliations:** 1 School of Physics, Changchun University of Science and Technology, Changchun 130022, China; zhouguanlong0104@163.com; 2 Optoelectronics System Laboratory, Institute of Semiconductors, Chinese Academy of Sciences, Beijing 100083, China; zhouyan@semi.ac.cn

**Keywords:** refractive index, position-sensitive detector (PSD), optical measurement, depth

## Abstract

The refractive index of seawater is one of the essential parameters in ocean observation, so it is necessary to achieve high-precision seawater refractive index measurements. In this paper, we propose a method for measuring the refractive index of seawater, based on a position-sensitive detector (PSD). A theoretical model was established to depict the correlation between laser spot displacement and refractive index change, utilizing a combination of a position-sensitive detector and laser beam deflection principles. Based on this optical measurement method, a seawater refractive index measurement system was established. To effectively enhance the sensitivity of refractive index detection, a focusing lens was incorporated into the optical path of the measuring system, and simulations were conducted to investigate the impact of focal length on refractive index sensitivity. The calibration experiment of the measuring system was performed based on the relationship between the refractive index of seawater and underwater pressure (depth). By measuring laser spot displacement at different depths, changes in displacement, with respect to both refractive index and depth, were determined. The experimental results demonstrate that the system exhibits a sensitivity of $9.93\times 10^{-9}\;RIU$ (refractive index unit), and the refractive index deviation due to stability is calculated as $\pm 7.54\times 10^{-9}\;RIU$. Therefore, the feasibility of this highly sensitive measurement of seawater refractive index is verified. Since the sensitivity of the refractive index measurement of this measurement system is higher than the refractive index change caused by the wake of underwater vehicles, it can also be used in various applications for underwater vehicle wake measurement, as well as seawater refractive index measurement, such as the motion state monitoring of underwater navigation targets such as AUVs and ROVs.

## 1. Introduction

Ocean parameters, such as temperature, salinity, and pressure of seawater, are important for the study of marine environment and flow field analysis. The traditional method of detecting the temperature, conductivity, and pressure of seawater is a CTD (conductivity–temperature–depth) device. Currently, a CTD can measure the temperature of seawater to a sensitivity of 0.001 °C. However, due to the limitations of this measurement method, CTD devices cannot detect non-ionic substances (SiO_2_, CO_2_, and soluble organics) in seawater [1,2]. This leads to deviations in salinity measurements.

Under this circumstance, measuring the refractive index using an optical method is an advantageous solution. The refractive index optical measurement is highly sensitive and has significance in the analysis of density [3], pressure [4,5], salinity [6,7], temperature [8,9], and the environment of the ocean [10]. In 2003, Y. Zhao et al. proposed an optical fiber sensor for the remote monitoring of salinity in water. This sensor was based on the detection of beam deviation, due to the refractive index changes in seawater, and salinity was measured using a PSD to a resolution of 0.012 g kg^−1^, and the method was applied in a laboratory environment [11]. In 2009, D. Malardé et al. proposed a compact optical refractometer, named NOSS, to measure the refractive index of seawater. This refractometer was based on the principle of light refraction, and it was developed using a V-block optical structure. The prototype is capable of measuring the seawater refractive index to a resolution of $4\times 10^{-7}\;RIU$, and it has already been applied to the in situ detection of the ocean refractive index [12,13]. In 2011, Y. C. Kim et al. developed a sensor of refractive index change based on combination surface plasmon resonance (SPR), which utilized the principle of the surface plasma effect between the evanescent wave on the optical fiber surface and the metal film coating. The type of fiber optic SPR sensor used here has a sensitivity of approximately 1600 nm/RIU and there is a refractive index change of $2\times 10^{-4}\;RIU/ppt$, with the limit of detection, i.e., the smallest shift detectable by the SPR spectrometer employed, being 0.04 nm [14]. This method has already been applied to the detection of ocean salinity. In 2016, Y. Hen et al. designed a sensor based on the modulation principle of fiber Bragg grating (FBG) for a broad-spectrum light source, which calculated the change in refractive index using wavelength bias. The standard deviation of the measured value of refractive index change was $1.46\times 10^{-4}\;RIU$ [15]. In 2018, J. Chen et al. developed a compact refractive index sensor based on the total internal reflection (TIR) method, with a sampling frequency of 0.1 Hz and a standard deviation of $4.78\times 10^{-6}\;RIU$ in the results obtained [16]. In 2018, Y. Zhao et al. developed a new sensing method for the simultaneous measurement of seawater temperature and salinity using C-type micro-structured fiber, with a maximum salinity sensitivity of 1.402 nm/‰ obtained for X-polarization and a maximum temperature sensitivity of 7.609 nm/°C obtained for Y polarization [17]. In 2019, H. Uchida et al. developed a state-of-the-art density sensor for seawater measurements with a sampling frequency of 1.2 Hz, which was based on measuring the refractive index using the interference method. The resolution of the density sensor is 0.00006 kg/m^3^ for changing temperatures at a constant salinity and pressure, 0.00012 kg/m^3^ for changing salinities at a constant temperature and pressure, and 0.00010 kg/m^3^ for changing pressures at a constant temperature and salinity. This method was suitable for refractive index measurements on marine samples in a laboratory environment [3]. In 2021, Y. Wang et al. proposed a fiber optic refractive index sensor, based on the anti-resonant reflecting optical waveguide and mode interference. The refractive index sensitivity of this sensor was 19,014.4 nm/RIU [18]. In 2021, C. R. Uma Kumari et al. proposed an evanescent wave absorbance (EWA) sensor in reflective mode, and the fabricated ER-EWA sensor measured changes in the RI of a seawater sample, with a sensitivity of 48.036 arbitrary units per RI unit (a.u./RIU), in the range of 1.332 RIU to 1.344 RIU [19]. In 2022, Xiaoguang Mu et al. proposed a fluorine-doped fiber (FDF)-sandwiched Mach–Zehnder interferometer (MZI), the RI sensitivity of which could be up to 14,638 nm/RIU. In contrast, the temperature cross-sensitivity of this sensor is only $1.7\times 10^{-6}\;RIU/{}^{\circ}C$ [20]. In 2022, F. Wu et al. designed a refractive index sensor based on the dramatic ellipsometric phase change at the long-wavelength band edge in an all-dielectric 1-D PhC. Assisted by the dramatic ellipsometric phase change at the long-wavelength band edge, the minimal resolution of the designed sensor reached $9.28\times 10^{-8}\;RIU$. This refractive index measurement method was suitable for monitoring the temperature, humidity, pressure, and concentration of biological analytes in a laboratory environment [21]. In 2023, G. Li et al. proposed a seawater salinity sensor array, based on a micro/nanofiber Bragg grating (MNFBG) structure, for which the salinity sensitivity for the two cascaded sensor arrays was 8.39 pm/‰ and 7.71 pm/‰, while the temperature sensitivity was 8.28 pm/°C and 8.03 pm/°C. This method was suitable for refractive index measurements of marine samples in a laboratory environment [22,23]. In 2023, Y. Wang proposed a sensor for the simultaneous measurement of seawater salinity and temperature, based on surface plasmon resonance (SPR) and Mach–Zehnder interference (MZI), with the sensitivity of seawater temperature being approximately 316.72 pm/°C with the temperature changing from 20 °C to 45 °C, and the sensitivity of seawater salinity being 429.72 pm/‰ [24].

According to the existing refractive index measurement requirements, the above refractive index optical measurement methods are compared and analyzed. At present, the sensitivity of the salinity measurement, based on detection using the beam deviation method, is 0.012 g kg^−1^, which is equivalent to a seawater refractive index of approximately $2.4\times 10^{-6}\;RIU$. The refractive index sensitivity of the NOSS is $4\times 10^{-7}\;RIU$. By our calculations, the refractive index sensitivity of the sensor of the SPR is $2.5\times 10^{-5}\;RIU$, and we obtained a measurement result with a standard deviation of $8.3\times 10^{-6}\;RIU$. By our calculations, the refractive index sensitivity of the sensor of the FBG is approximately $10^{-4}\;RIU$. The refractive index sensitivity of the sensor of the TIR is approximately $10^{-5}\;RIU$, and we obtained a measurement result with a standard deviation of $4.78\times 10^{-6}\;RIU$. The sensitivity of the refractive index measurement based on the interference method is $1.33\times 10^{-7}\;RIU$, and the standard deviation of the sensor refractive index is $2.93\times 10^{-8}\;RIU$. The sensitivity of the refractive index measurement based on the anti-resonant reflecting optical waveguide and mode interference is approximately $2.1\times 10^{-6}\;RIU$. The sensitivity of the refractive index measurement based on the dramatic ellipsometric phase change at the long-wavelength band edge in an all-dielectric 1-D PhC is $9.28\times 10^{-8}\;RIU$. The sensitivity of the refractive index measurement based on MNFBG is approximately $10^{-8}\;RIU$.

The currently available refractive index sensors play a great role in the fields of seawater quality monitoring, the establishment of temperature and salinity depth structure of seawater, and the analysis of currents at the mouth of the sea. However, in the field of ocean currents and ocean climate research, the refractive index change per 100 km in the western North Pacific is $4\times 10^{-8}\;RIU$ [3], which is beyond the resolving capability of most refractive index sensors. In the field of seawater flow field monitoring, the study of the wave number spectrum of Batchelor turbulence with a refractive index gradient [25] shows that strong refractive index signal variations occur in the high-frequency band within a certain wave number interval, which puts demands on the sampling frequency of the sensor. Simulation studies of submarines [26] have shown that the time grid for simulation calculations needs to be of the order of 0.01 s or even a few milliseconds to obtain converged high-precision simulation results, where the amount of refractive index change in the fine structure is of the order of $10^{-8}\;RIU$. Thus, the sensitivity of the refractive index measurement based on the MNFBG meets the technical requirements of marine monitoring, while the sensitivity of the other measurement methods cannot meet the accuracy needed for the measurement of the ocean refractive index. However, the MNFBG refractive index measurement methods make it relatively easy to achieve refractive index measurements under good laboratory conditions, but are not suitable for harsh field experimental conditions, such as complex oceans.

Based on these new application requirements, we proposed a method for measuring the refractive index of seawater, based on a PSD. A theoretical model was established to depict the correlation between laser spot displacement and refractive index change, utilizing a combination of a position-sensitive detector and laser beam deflection principles. Based on this optical measurement method, a seawater refractive index measurement system was established. We improved the sensitivity of the seawater refractive index measurement by improving the structure and design of the optical path of the seawater refractive index measurement system. A focusing lens was incorporated into the optical path of the measuring system, and simulations were conducted to investigate the impact of focal length on refractive index sensitivity. Considering the sensitivity and compactness of the system, the length of the measurement area of the system was determined to be *L* = 100 mm, and the focal length of the focusing lens of the system was determined to be *f* = 300 mm. The calibration experiment of the measuring system was performed based on the relationship between the refractive index of seawater and underwater pressure (depth). By measuring laser spot displacement at different depths, changes in displacement, with respect to both refractive index and depth, were determined. The experimental results demonstrated that the system exhibited a sensitivity of $9.93\times 10^{-9}\;RIU$, and the refractive index deviation due to stability was calculated as $\pm 7.54\times 10^{-9}\;RIU$. Therefore, the feasibility of this highly sensitive measurement of seawater refractive index was verified.

## 2. Structure and Principle of the Refractive Index Optical Measurement System

**Figure 1:**
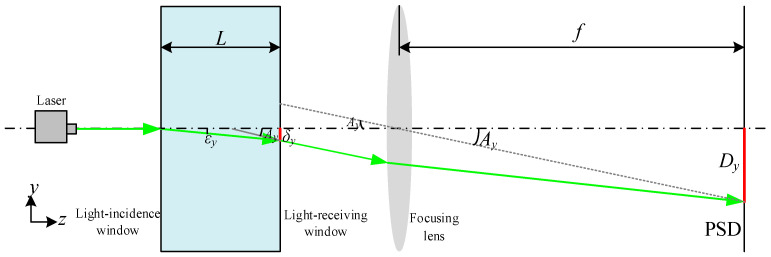
Schematic diagram of seawater refractive index measurement based on the laser beam deviation technique.

The optical seawater refractive index measurement adopts the laser beam deviation technique, and its schematic diagram is shown in Figure 1. The light emitted from the laser passes through the light-incidence window, and it reaches the seawater refractive index measurement area. Due to the refractive index gradient of seawater, the laser–seawater interaction causes a laser refraction phenomenon. Then, the laser passes through the light-receiving window, and the laser is gathered to the spot on the photosensitive surface of the PSD by the focusing lens. Thus, the laser spot position coordinates are obtained using the PSD.

The geometric theory of light refraction is an approximation of the physical optics approach, assuming that the measurement system optical path is a two-dimensional optical path that is isotropic in the *x* and *y* directions. Since it is difficult to ensure that the incident laser and the light-receiving window are perfectly perpendicular, the optical path is deflected by the refractive index gradient in the *y*-direction when the laser enters the measurement zone, so only the refractive index gradient in the *y* direction is considered. The refraction of light caused by the inhomogeneity of light is proportional to the gradient of refractive index in each direction in the *y* planes. Therefore, the deflection rate of light rays can be expressed as in [27], as follows:

(1) $$\frac{\partial^{2}y}{\partial z^{2}}=\frac{1}{n}\frac{\partial n}{\partial y}$$

where *n* is the refractive index, *z* is the direction of light propagation, and the curvature of the refracted light $\frac{\partial^{2}y}{\partial z^{2}}$ is represented by the gradient of the refractive index $\frac{\partial n}{\partial y}$ of seawater.

In the refractive index measurement area, the angle of deflection of the light ray in the direction of *y* axes is obtained by integrating Equation (1), as follows:

(2) $$\varepsilon_{y}=\frac{1}{n}\int \frac{\partial n}{\partial y}\partial z$$

where the $\varepsilon_{y}$ is the angle of deflection of the light ray in the direction of *y* axis and $\frac{\partial n}{\partial y}$ is the gradient of the refractive index in the *y* axis direction.

Since the deflection angle is small and the distance between the light-incidence window and the light-receiving window is *L*, there is the following relationship:

(3) $$\varepsilon_{y}=\frac{L}{n_{0}}\frac{\partial n}{\partial y}\approx \tan\varepsilon_{y}$$

where *n*_0_ is the refractive index of the surrounding seawater.

Therefore, the gradient of the refractive index in the *y* axis direction is expressed as follows:

(4) $$\frac{\partial n}{\partial y}=\tan\varepsilon_{y}\frac{n_{0}}{L}=\frac{{\delta_{y}n}_{0}}{L^{2}}$$

where the $\delta_{y}$ is the displacement of the light ray in the direction of *y* axis.

The relationship between the displacement $\delta_{y}$ generated in the refractive index measurement area and the displacement $D_{y}$ on the PSD photosensitive surface is shown in Figure 1. If the refractive index measurement zone distance *L* is short enough, the spot position point on the light-receiving window can be approximated as the corresponding seawater refractive index. The law of refraction is satisfied at the light-receiving window. Therefore, the following are true:

(5) $$\sin\varepsilon_{y}=\frac{\delta_{y}}{\sqrt{\delta_{y}^{2}+L^{2}}}$$

(6) $$\sin A_{y}=\frac{D_{y}}{\sqrt{D_{y}^{2}+f^{2}}}$$

(7) $$n_{1}\sin A_{y}=\left(n_{0}+\delta_{y}\frac{\partial n}{\partial y}\right)\sin\varepsilon_{y}$$

where $n_{1}$ is the refractive index of air, $A_{y}$ is the spatial angle from the center of the focusing lens to the spot position on the PSD photosensitive surface in the direction of the *y* axis, *f* is the focal length of the focusing lens, and $\left(n_{0}+\delta_{y}\frac{\partial n}{\partial y}\right)$ is the measured refractive index of the seawater.

Thus, by association, Equations (4)–(7) are obtained as follows:

(8) $$\frac{\delta_{y}\left(\delta_{y}^{2}+L^{2}\right)}{\sqrt{\delta_{y}^{2}+L^{2}}}=\frac{L^{2}D_{y}}{n_{0}\sqrt{D_{y}^{2}+f^{2}}}$$

Equation (8) is obtained by substituting the trigonometric relations, Equations (5) and (6), and the y-direction refractive index gradient, Equation (4), into the law of refraction, Equation (7).

Substituting Equation (8) into Equation (4), it is expressed as follows:

(9) $$\frac{\partial n}{\partial y}=\frac{D_{y}}{\sqrt{D_{y}^{2}+f^{2}}\sqrt{\delta_{y}^{2}+L^{2}}}$$

Since the focal length *f* is much larger than $D_{y}$ and the length of the measuring zone *L* is much larger than $\delta_{y}$, there exists the following:

(10) $$\frac{\partial n}{\partial y}=\frac{D_{y}}{fL}$$

The measured refractive index of the seawater can be expressed as follows:

(11) $$n=n_{0}+\delta_{y}\frac{D_{y}}{fL}$$

**Figure 2:**
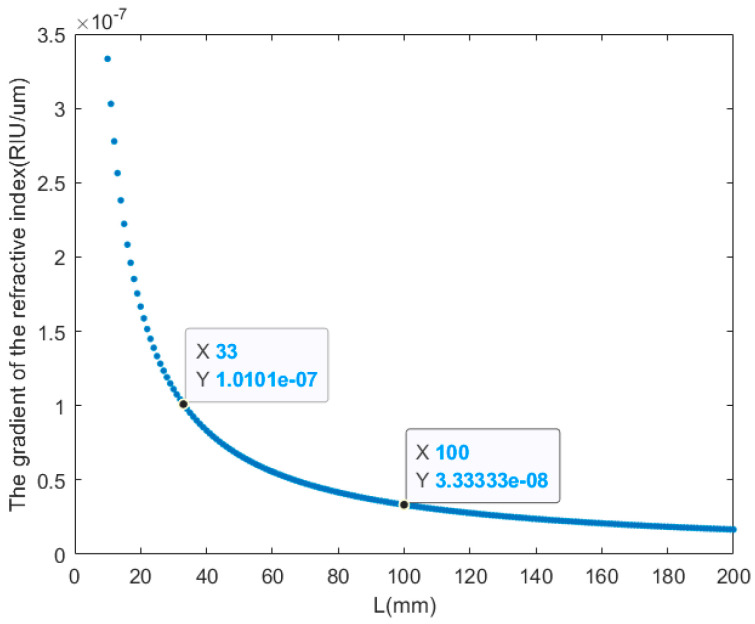
Relationship between the gradient of the refractive index and the length of the measurement area *L*.

The relationship between the gradient of the refractive index of the refractive index measurement system and the length of the measurement area *L* is shown in Figure 2. Considering the sensitivity and compactness of the system, the focal length of the focusing lens of the refractive index system is determined to be f=300 mm, the sensitivity of the PSD is $D=10^{-3}\;mm$, and the length range of the measurement area is set to be 10 mm≤L≤200 mm. When 10 mm≤L≤33 mm, the sensitivity of the refractive index measurement system is of the order of $10^{-7}\;RIU$. When 33 mm<L≤200 mm, the sensitivity of the refractive index measurement system is of the order of $10^{-8}\;RIU$. Considering the sensitivity and stability of the refractive index measurement of the system, the length of the measurement area of the system is determined to be L=100 mm, and the sensitivity of the refractive index measurement of the system is $3.33\times 10^{-8}\;RIU$. The refractive index measurement sensitivity changes by $3\times 10^{-10}\;RIU$ for every 1 mm change in the length of the measurement area.

**Figure 3:**
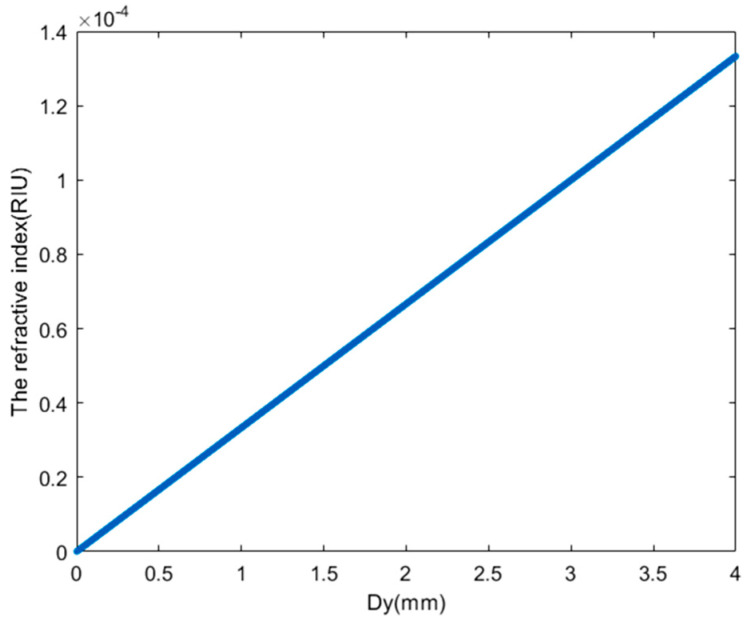
Relationship between the refractive index change of the refractive index measurement system and the laser spot position change $D_{y}$.

The relationship between the refractive index change of the refractive index measurement system and the laser spot position change $D_{y}$ is shown in Figure 3. When the length of the measurement area L=100 mm and the focal length range of the focusing lens f=300 mm, the laser spot position change is ${0.001\;mm\leq D}_{y}\leq 4\;mm$. The range of the refractive index change measurement of the system is from $3.33\times 10^{-8}\;RIU$ to $1.33\times 10^{-4}\;RIU$.

## 3. Establishment of the Optical Measurement System and Experimental Results

### 3.1. Establishment of the Seawater Refractive Index Measurement System

**Figure 4:**
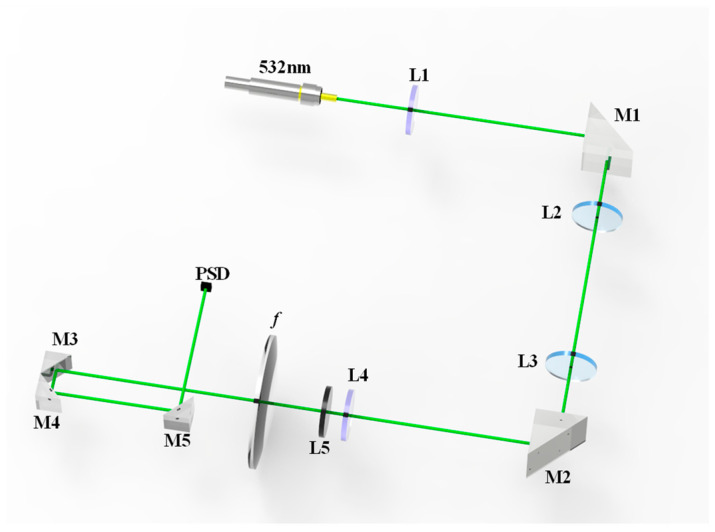
Optical configuration of the seawater refractive index measurement system based on a PSD.

The seawater refractive index optical measurement system is based on a PSD (TEM Messtechnik GmbH, Hannover, Germany), and its optical configuration diagram is shown in Figure 4.

**Figure 5:**
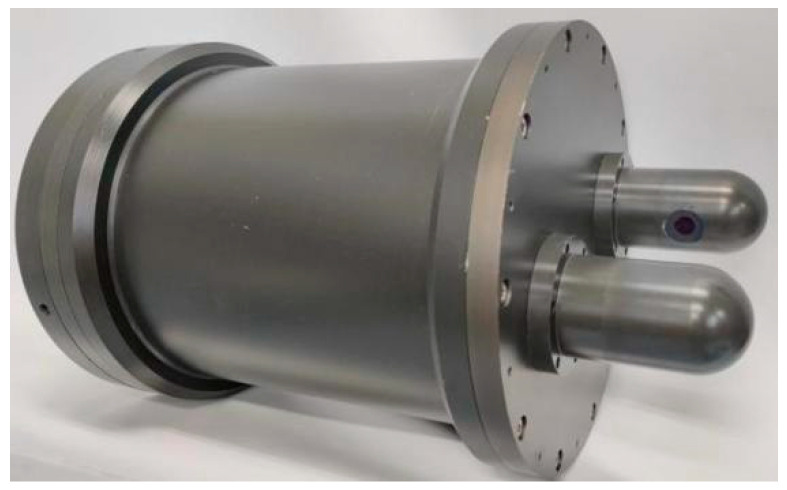
Seawater refractive index optical measurement system based on a PSD.

With a view to investigating the absorption and scattering effects in the seawater, the optical system used a green collimated laser with a central wavelength of 532 nm as the measurement light source. After the laser passes through lens L1, the right-angle prism M1 is used to reflect the incident beam and the light-incidence window L2, it reaches the seawater refractive index detection area. After the laser–seawater interaction causes a laser refraction phenomenon, the laser passes through the light-receiving window L3, the right-angle prism M2, and then the lens L4. In front of the light-sensitive surface of the PSD, the narrow-band light filter M5, with the same peak wavelength and a bandwidth of 1.1 nm, is used to filter out the stray light, except for the 532 nm measurement light. The laser is gathered to the spot on the photosensitive surface of the detector by the focusing lens *f,* and a laser spot diameter of 0.12954 mm is focused onto the PSD photosensitive surface. In order to realize the miniaturization of the measurement system structure, right-angled prisms M3, M4, and M5 are added between the focusing lens and the detector’s photosensitive surface, thereby changing the laser transmission direction. The dimensions of the PSD’s photosensitive area are 4 mm×4 mm, and the position measurement resolution is approximately 1 μm. Based on the transverse photoelectric effect, the PSD’s output signal is independent of the incident light intensity and is only related to positions of the incident beam. The seawater refractive index optical measurement system based on a PSD is shown in Figure 5.

### 3.2. Experimental Setup of the Calibration Experiment

**Figure 6:**
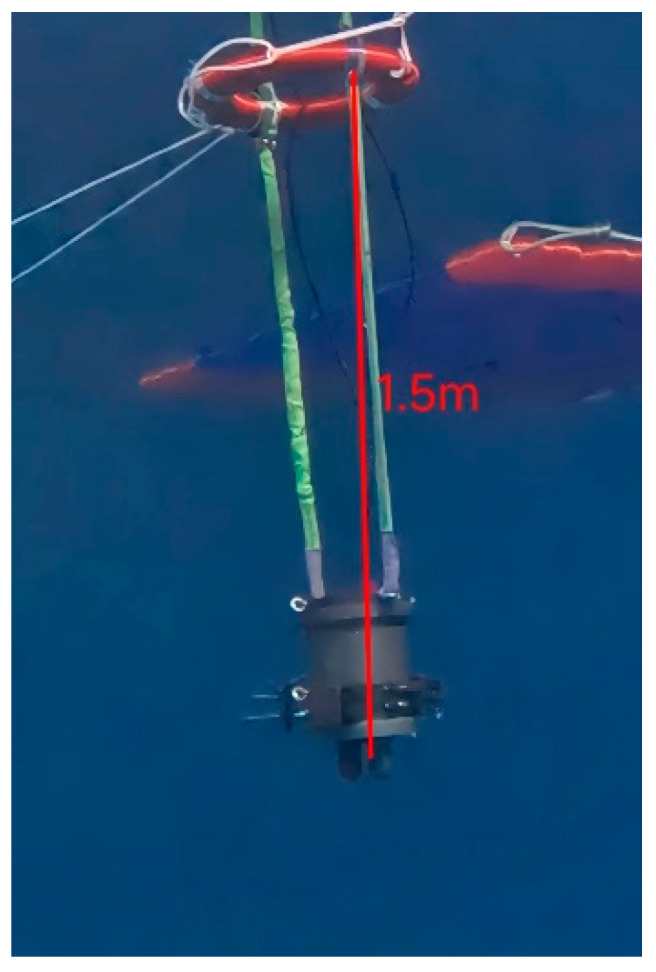
Experimental scenario of the calibration experiment.

The calibration experiment of the seawater refractive index optical measurement system was carried out in an indoor pool with constant temperature and salinity, and the experimental scenario is shown in Figure 6. The seawater refractive index measurement system is mounted upside down and fixed to the stepper motor, and the refractive index measurement zone is placed approximately 1.5 m underwater. The seawater refractive index measurement system is immersed in the seawater to be measured for 1 h, in order to make its temperature the same as the temperature of the seawater to be measured, thus reducing the measurement errors generated by the temperature. The different laser spot positions on the PSD photosensitive surface, corresponding to different depths underwater, are obtained by this optical measurement system. The seawater refractive index optical measurement system has a sampling frequency of 10 kHz and a sampling time of approximately 60 s. The seawater refractive index measurement system is lifted a distance of approximately 3 cm, changing the submersion depth and thus the pressure in the detection area, and data are recorded for approximately 60 s after each change in underwater depth. The experiment is concluded after recording 30 sets of refractive index measurements.

### 3.3. Experimental Results of the Calibration Experiment

**Figure 7:**
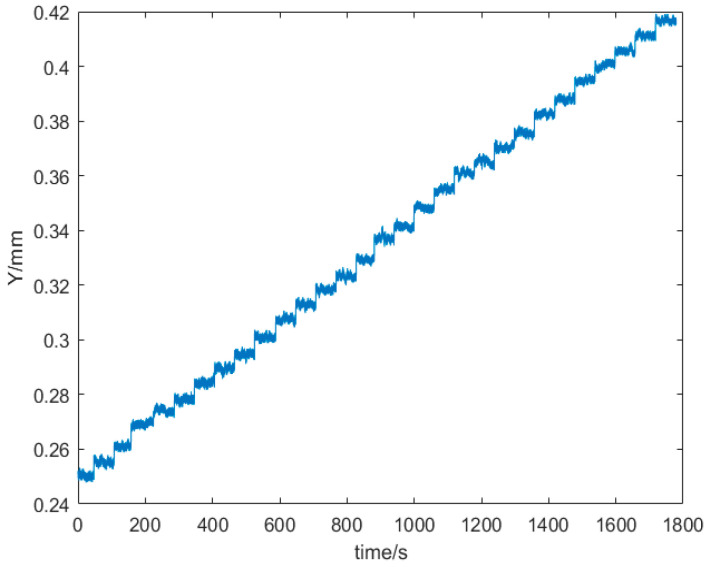
Time domain diagram of the experimental results of the calibration experiment of the seawater refractive index optical measurement system.

The time domain diagram of the experimental result of the calibration experiment of the seawater refractive index measurement system is shown in Figure 7.

According to the experimental results, since the underwater depth of the seawater refractive index measurement system varied with time and stayed at each depth for approximately 60 s, the time–position line is stepped. The laser spot moves from a position of 0.24277 mm to 0.41671 mm on the PSD photosensitive surface. On average, each depth change corresponded to a laser spot position change of 0.006 mm. The theoretical calculations yielded a 0.006 mm displacement change of the laser spot, corresponding to a refractive index change of $1.99\times 10^{-7}\;RIU$.

**Figure 8:**
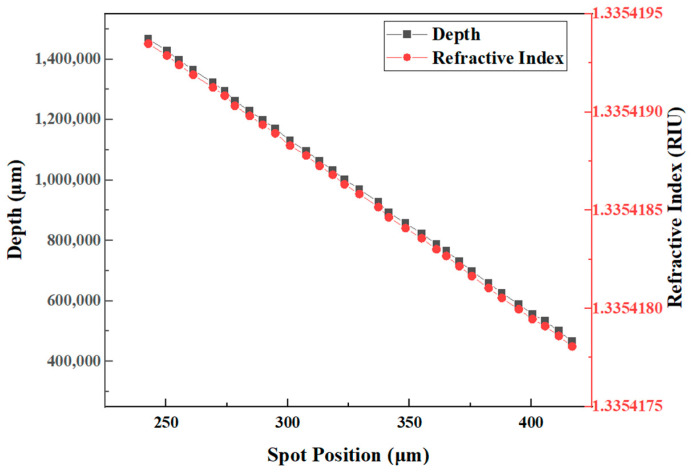
Linear fit plot of depth with spot positions and refractive index with spot positions.

The laser spot position on the PSD photosensitive surface varied linearly as the measurement system changes depth underwater. The 60 s’ worth of experimental data obtained from different underwater depth measurements were averaged separately. Linear fitting was established, based on the proportional relationship between underwater depth and pressure and the variation in refractive index with pressure, as expressed by TEOS–10 (2010 Thermodynamic Equation of Seawater) [28]. Thus, the underwater depth changed by 0.01 dbar per 1 cm change in pressure, and the underwater pressure changed by $1.54\times 10^{-8}\;RIU$ per 0.01 dbar change in refractive index. Experimental results of the calibration experiment of the seawater refractive index optical measurement system shown in Table A1. The linear fit plot of depth with spot positions and refractive index with spot positions are shown in Figure 8. Both the underwater depth and the refractive index were linearly correlated with the spot positions and followed the same trend. The fit of the gradient of refractive index of the seawater refractive index optical measurement system was $9.93\times 10^{-9}\;RIU/\mu m$. Since the detection sensitivity of PSD is 1 μm, the sensitivity of the optical measurement system for the refractive index of seawater was $9.93\times 10^{-9}\;RIU$. Due to the high sensitivity of the system measuring the refractive index of seawater, there can be a signal jitter or measurement errors in the measurement signal, due to the presence of small changes in temperature and other parameters of the seawater sample. Theoretical calculations yielded a 0.006 mm displacement change of the laser spot, corresponding to a refractive index change of $5.96\times 10^{-8}\;RIU$. The theoretically calculated value was 3.36 times the calculated value of the fitted relationship. The experimental results of the calibration of the seawater refractive index measurement system outperformed the results of the numerical simulation.

### 3.4. Experimental Setup of the Refractive Index Measurement Experiment

**Figure 9:**
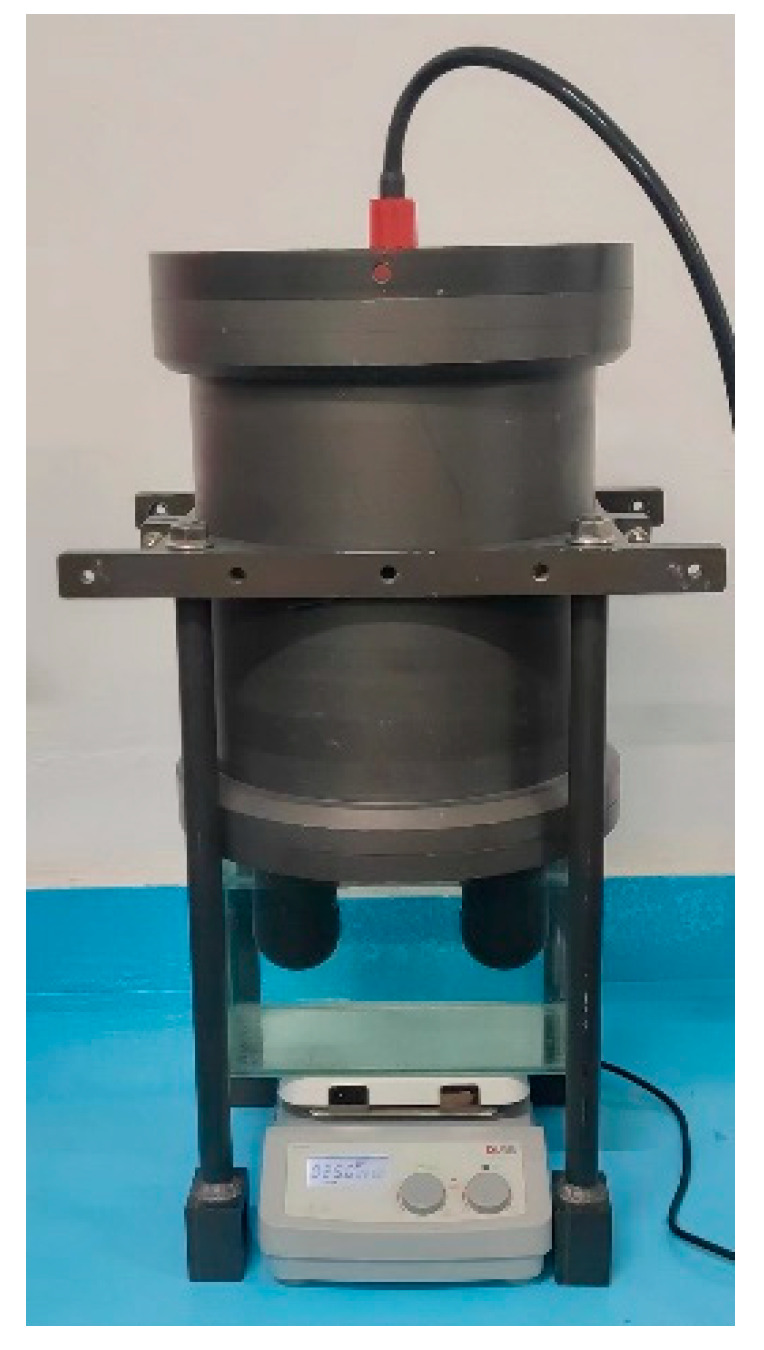
Experimental setup for the standard salinity seawater measurement experiment.

The seawater refractive index measurement experiment with standard salinity seawater was carried out in an indoor glass water cylinder with constant temperature and pressure levels, and the experimental scenario is shown in Figure 9.

The seawater refractive index measurement system was mounted upside down, and the seawater refractive index detection area was immersed in standard refractive index seawater. The experiments were carried out using standard seawater from the National Centre for Marine Standards and Metrology of China for refractive index measurement experiments. In the experiments, the temperature of the seawater samples was controlled to be 25 °C, the laboratory had a standard atmospheric pressure of 10.1325 dbar, and the ambient humidity was 60%. The refractive index of standard salinity seawater is shown in Table 1.

### 3.5. Experimental Results of the Refractive Index Measurement Experiment

**Figure 10:**
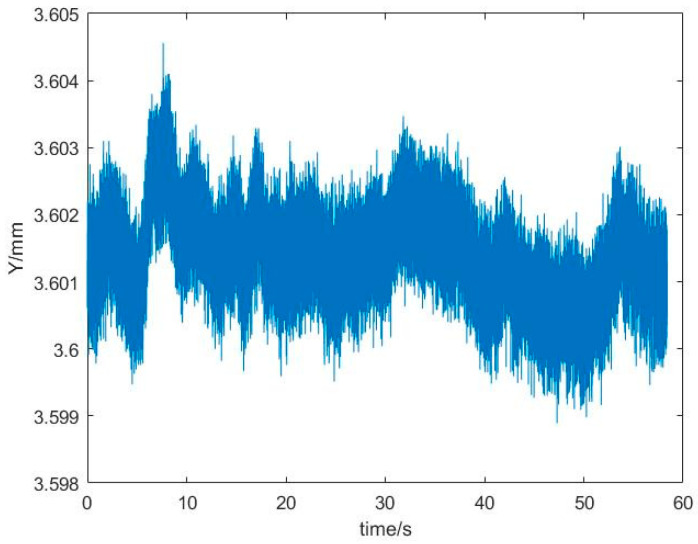
Stability of the seawater refractive index measurement system.

The results of the seawater refractive index measurement experiment with standard salinity seawater are shown in Figure 10. The sampling frequency of the seawater refractive index measurement system was 5 kHz, and the sampling time was approximately 60 s. The average value of the laser spot position data for this experiment was 3.6014 mm. According to the fitting relationship, when the refractive index of standard salinity seawater is 1.3353862, the position of the laser spot should be 3.603 mm. Thus, the laser spot position deviation is 0.0016 mm, corresponding to a refractive index deviation of $1.59\times 10^{-8}\;RIU$.

## 4. Discussion

The stability of the seawater refractive index measurement system is an important indicator of reliability and reflects the accuracy of the system’s measurements. According to the results of the seawater refractive index measurement experiment with standard salinity seawater, the jitter signal of the system was ±0.00176 mm. Based on the measurement sensitivity of the system, the refractive index deviation, due to stability, was calculated as $\pm 7.54\times 10^{-9}\;RIU$, since the system refractive index measurement sensitivity was $9.93\times 10^{-9}\;RIU$. The refractive index deviation of the seawater refractive index measurement system was less than the refractive index measurement sensitivity and was within the error range. Additionally, these results are better than those obtained by Hiroshi Uchida et al., who obtained seawater refractive index measurements with a standard deviation of $2.93\times 10^{-8}\;RIU$, using the optical measurement technique [3]. Therefore, the refractive index deviation, due to the jitter signal, does not significantly affect the accuracy of the final refractive index measurement. The cause of signal jitter in the experimental results could be the fluctuation of seawater temperature in the measurement area. Although we tried to minimize the measurement errors caused by the design and construction of the optical structure, the resulting refractive index measurement errors still exist, mainly due to errors in the length of the measurement zone and the angle at which the optics, such as lenses, are mounted, during the construction of the system.

## 5. Conclusions

In this paper, we proposed a method for measuring the refractive index of seawater based on a PSD. A theoretical model was established to depict the correlation between laser spot displacement and refractive index change, utilizing a combination of a position-sensitive detector and laser beam deflection principles. Based on this optical measurement method, a seawater refractive index measurement system was established. To effectively enhance the sensitivity of refractive index detection, a focusing lens was incorporated into the optical path of the measuring system, and simulations were conducted to investigate the impact of focal length and the length of the measurement area on refractive index sensitivity. Considering the sensitivity and compactness of the system, the length of the measurement area of the system was determined to be *L* = 100 mm, and the focal length of the focusing lens of the system was determined to be *f* = 300 mm. The calibration experiment of the measuring system was performed based on the relationship between refractive index of seawater and underwater depth. By measuring laser spot displacement at different depths, changes in displacement, with respect to both refractive index and depth, were determined. The experimental results demonstrated that the fit of the gradient of the refractive index of the optical measurement system was $9.93\times 10^{-9}\;RIU/\mu m$, and since the detection sensitivity of a PSD is 1 μm, the system exhibited a sensitivity of $9.93\times 10^{-9}\;RIU$. The stability of the seawater refractive index measurement system was measured by standard salinity seawater refractive index measurement experiments, and the result of the refractive index deviation, due to stability, was calculated as $\pm 7.54\times 10^{-9}\;RIU$. Therefore, the feasibility of this highly sensitive measurement of seawater refractive index was verified.

According to various comparisons, the existing seawater refractive index measurement methods do not give satisfactory results for marine refractive index gradient measurements, but the proposed method was proven to be feasible. Furthermore, the proposed seawater refractive index measurement system did not require complex optical structures. Since the sensitivity of the refractive index measurement of this measurement system was higher than the refractive index change caused by the wake of underwater vehicles, it can also be used in various applications of underwater vehicle wake measurement, as well as seawater refractive index measurement, such as motion state monitoring of underwater navigation targets such as AUVs and ROVs. In our future works, we will apply the proposed seawater refractive index measurement system to measure the refractive index of different marine environments, such as different sea areas and different ocean depths.

## Figures and Tables

**Table 1:** Refractive index of standard salinity seawater.

Salinity (PSU)	Temperature (°C)	Pressure (dbar)	Humidity	Refractive Index (RIU)
20.009	25.00	10.1325	60%	1.3353862

## Data Availability

Data are contained within the article.

## Appendix A

**Table A1:** Experimental results of the calibration experiment of the seawater refractive index optical measurement system.

Spot Position	Depth	Refractive Index
(μm)	(μm)	(RIU)
242.71	1,467,100	1.3353193483
250.36	1,428,000	1.3353192881
255.41	1,396,700	1.3353192399
261.17	1,364,100	1.3353191897
269.31	1,322,100	1.3353191250
274.14	1,294,900	1.3353190831
278.26	1,261,500	1.3353190317
284.36	1,228,300	1.3353189805
289.66	1,199,000	1.3353189354
294.85	1,170,660	1.3353188918
300.97	1,129,800	1.3353188289
307.58	1,097,000	1.3353187783
313.06	1,063,000	1.3353187260
318.47	1,033,400	1.3353186804
323.31	1,002,100	1.3353186322
329.43	969,900	1.3353185826
337.22	926,600	1.3353185159
341.46	893,100	1.3353184643
348.38	856,900	1.3353184086
354.98	823,800	1.3353183576
361.09	787,100	1.3353183011
365.14	765,300	1.3353182675
370.45	731,500	1.3353182155
375.6	698,600	1.3353181648
382.5	659,200	1.3353181041
387.93	626,400	1.3353180536
394.88	588,900	1.3353179959
400.49	556,300	1.3353179457
405.68	533,200	1.3353179101
411.3	500,700	1.3353178600
416.77	465,800	1.3353178063
